# Targeting of mesenchymal stem cells to ovarian tumors via an artificial receptor

**DOI:** 10.1186/1757-2215-3-12

**Published:** 2010-05-25

**Authors:** Svetlana Komarova, Justin Roth, Ronald Alvarez, David T Curiel, Larisa Pereboeva

**Affiliations:** 1 Division of Human Gene Therapy, Department of Medicine, University of Alabama at Birmingham, Birmingham, Alabama 35294- 2172, USA; 2 Division of Human Gene Therapy, Department of Pathology, University of Alabama at Birmingham, Birmingham, Alabama 35294- 2172, USA; 3 Division of Human Gene Therapy, Department of Surgery, University of Alabama at Birmingham, Birmingham, Alabama 35294- 2172, USA; 4 Division of Human Gene Therapy, the Gene Therapy Center, University of Alabama at Birmingham, Birmingham, Alabama 35294- 2172, USA; 5 The Division of Gynecologic Oncology, Department of Obstetrics and Gynecology, University of Alabama at Birmingham, Birmingham, AL 35213, USA

## Abstract

**Background:**

Mesenchymal Progenitor/Stem Cells (MSC) respond to homing cues providing an important mechanism to deliver therapeutics to sites of injury and tumors. This property has been confirmed by many investigators, however, the efficiency of tumor homing needs to be improved for effective therapeutic delivery. We investigated the feasibility of enhancing MSC tumor targeting by expressing an artificial tumor-binding receptor on the MSC surface.

**Methods:**

Human MSC expressing an artificial receptor that binds to erbB2, a tumor cell marker, were obtained by transduction with genetically modified adenoviral vectors encoding an artificial receptor (MSC-AR). MSC-AR properties were tested *in vitro *in cell binding assays and *in vivo *using two model systems: transient transgenic mice that express human erbB2 in the lungs and ovarian xenograft tumor model. The levels of luciferase-labeled MSCs in erbB2-expressing targeted sites were evaluated by measuring luciferase activity using luciferase assay and imaging.

**Results:**

The expression of AR enhanced binding of MSC-AR to erbB2-expressing cells *in vitro*, compared to unmodified MSCs. Furthermore, we have tested the properties of erbB2-targeted MSCs *in vivo *and demonstrated an increased retention of MSC-AR in lungs expressing erbB2. We have also confirmed increased numbers of erbB2-targeted MSCs in ovarian tumors, compared to unmodified MSC. The kinetic of tumor targeting by ip injected MSC was also investigated.

**Conclusion:**

These data demonstrate that targeting abilities of MSCs can be enhanced via introduction of artificial receptors. The application of this strategy for tumor cell-based delivery could increase a number of cell carriers in tumors and enhance efficacy of cell-based therapy.

## Background

In the last few years, cells have been increasingly used as vehicles for the delivery of therapeutics. The cell-based approach is particularly attractive for the delivery of biotherapeutic agents that are difficult to synthesize, have limited tissue penetrance, or are rapidly inactivated upon direct *in vivo *introduction. Some of the key factors for the success of this type of therapeutic delivery have been established, such as the means and efficiency of cell loading with a therapeutic payload, and the nature of therapeutics that the cells can carry. However, the issue of biodistribution of injected cell carriers *in vivo *still remains an important aspect of cell-based delivery that has yet to be fully investigated. Importantly, different types of cell vehicles may have specific biodistribution or cell homing patterns and, therefore, may provide a special advantage to achieve site-specific or targeted delivery of therapeutics.

The ability of injected cells to either passively concentrate in specific organs or actively home to disease sites supports the rationale for targeted delivery of therapeutics by cell vehicles. There is growing evidence that sites of injury or growing tumors favor active homing of endogenous and exogenous stem or progenitor cells [1,2]. The first observation of this phenomenon was published by Studeny et al, using MSCs as vehicles delivering IFNβ [3]. This and a subsequent study by the same group [4] reported MSC localization in lung tumors after systemic injection of these cells. The recognition that the tumor microenvironment or tumor cytokine profile is similar to that of inflammatory sites evoked a search for the tumor attracting signals. Despite still incomplete knowledge of these cues, the practical aspects of cell-based delivery of therapeutics to specific sites have been actively exploited. A growing number of studies have used MSCs as cell vehicles to exploit their native ability to target tumors, as a means to track malignant tissues or for the delivery of anticancer agents to tumors [2,5-9]. Several studies investigated MSCs as cell vehicles for the delivery of various clinically relevant anticancer factors, including cytokines, interferons, pro-drugs and replication competent adenoviruses, with noted benefits [10-13]. The native tumor homing phenomenon of MSCs was confirmed in different experimental systems [2,12]. Other cell types, such as umbilical cord matrix stem cells (UCMS) [14], neural stem cells [15,16] and endothelial progenitor cells [17,18] have also demonstrated the inherent ability to migrate toward tumors or other pathologies.

Along with using native cell homing properties, modification of the cell membrane by expressing appropriate receptors was also proposed as a means to obtain targeted cell vehicles. Much of the groundwork for such targeting approaches has previously been established for immune cells (T-cells, NK cells, CIK cells), where lymphocyte populations were modified to express artificial receptors (T-bodies) with distinct binding specificities to target cells. Artificial or chimeric receptors (AR) have been derived from the binding domains of antibodies (usually the single chain antibody, scFv) or T-cell receptors. An array of chimeric receptors, mostly with specificity for different tumor markers, has been tested for biological function *in vitro *[19,20] and *in vivo *[21,22]. This approach is often termed "targeted" adoptive immunotherapy, since the active targeting mechanism was added to redirect the native killing function of an immune cell to a defined target cell. Remarkably, the added affinity to retarget cell killing function was found to enhance localization of the modified cells to the target sites. Several studies demonstrated that AR-modified lymphocytes are detected in higher numbers in tumors that express the cognate receptor, compared to untargeted cells [23,24].

Despite showing its potential, the AR-based strategy has not been translated to other cell types that may serve as promising cell vehicles. Only a few applications have demonstrated the feasibility of using AR as a binding moiety in non-immune cell contexts [25,26]. In other examples, surface-expressed scFvs served as artificial receptors for viral infection [27] or enhanced the tumor cell binding [28]. Therefore, applying the AR strategy to other cell types and investigating the potential targeting benefits holds promise as a means to increase cell concentration in desired sites. Of note, most of the studies using native MSC homing did not quantitatively determine the level of cells that home to tumors or other sites. The tumor homing behavior of MSCs was demonstrated by the mere presence of these cells in the sites of interest and/or lack of such cells in other organs [8,11,13,29]. The few studies that did attempt to quantitatively estimate MSC numbers localized in tumors have reported low to moderate numbers [3,5,10]. Since increasing the number of cell vehicles in tumors would parallel therapeutic efficacy, investigation of native or artificial means of cell homing to tumors are of high therapeutic importance.

The present study tested the hypothesis that artificial receptors with affinities to target sites can be added to cell vehicles and the new cell binding properties can be utilized to increase cell vehicle levels in the target sites. Specifically, we investigated the possibility of increasing the number of MSCs in ovarian tumors by expressing a tumor antigen-binding receptor on the MSC surface. This would provide an additional means to increase the number of tumor-associated MSCs beyond their native tumor homing potential. To this end, we have created MSCs that express an artificial receptor (AR) that binds to erbB2, a frequent marker of tumor cells (MSC-AR). We have shown that these AR-expressing MSCs (MSC-AR) have enhanced binding to erbB2-expressing cells *in vitro*. Furthermore, we tested erbB-2 targeting of MSC-AR in model systems *in vivo *and demonstrated that addition of the AR increased retention of circulating MSC-AR in erbB2-expressing sites. We also confirmed an increased concentration of MSC-AR, compared to MSC, in erbB2 positive ovarian tumors.

These data show that the number of tumor-associated MSCs can be increased via affinity-based targeting, which can potentially serve to improve therapeutic delivery. Broadly, we demonstrated that an artificial cell targeting strategy can be beneficial to MSC-based cell vehicles and suggests that this strategy could also have potential for other cell types that lack native homing abilities.

## Methods

### Reagents

Anti-HA antibody conjugated to horse radish peroxidase (HRP) clone HA-7 (Sigma, Saint-Louis, Missouri) was used for detection of artificial receptor expressed on MSCs membrane. Anti-erbB2 (HER-2/neu) antibody, clone AM-2000-01 (Innogenex, San Ramon, CA) was used to test expression of the erbB2 protein. Goat anti-mouse IgG1 (HRP conjugated) was used as a secondary antibody (DAKO corporation, Carpinteria, CA). CFDA-CE and SP-DiI fluorescent dyes (green and red fluorescence correspondingly) for cell labeling were from Molecular Probe (Eugene, OR).

### Cell lines

The human ovarian carcinoma cell line SKOV3ip1 was obtained from Dr. Janet Price (University of Texas M.D. Anderson Center, Houston, TX). K562 cells - were obtained from ATCC the American Type Culture Collection (Manassas, VA) and cultured as recommended. Cells were maintained in DMEM/F-12, containing 10% fetal bovine serum (FBS) (HyClone, Logan UT) and 2 mM glutamine at 37°C in a humidified atmosphere of 5% CO_2_.

#### Isolation and culture of MSCs

Primary human MSCs were obtained from bone marrow draw leftovers (screen filters with bone marrow cells remaining) from several individuals undergoing bone marrow harvest for allogeneic transplantation at the UAB Stem Cell Facility under an approved IRB protocol. MSCs were isolated and cultured as previously described [30]. Cells were expanded by consecutive subcultivations in α-MEM with 10% FBS at densities of 5000-6000 cells/cm^2 ^and used for experiments at passages 2-8.

#### Recombinant adenoviruses

Adenoviral vectors having either wild type or genetically modified Ad5 fibers were used for experiments to load MSC with the targeting moiety and reporter genes. The following viruses were used in the study: AdCMV.AR, Ad.RGD.AR, Ad.RGDpK7.ARluc, Ad.RGDpK7.GFPluc. All viruses were replication-incompetent recombinant adenoviral (Ad) vectors having either single transgene or double cassette of transgenes in the E1 region under control of two CMV promoters. Coding sequences of AR, firefly luciferase and GFP were amplified by PCR from the plasmids pDisplayAR, pGL3 (Promega, Madison, WI) and pTrack (Qbiogene, Solon, OH) correspondingly and cloned into pShuttle plasmid.

AdCMV.AR has a wild type Ad5 fiber; AdRGD.AR has a fiber protein with an integrin binding motif (CDCRGDCFC) inserted in the HI loop [31]; both AdRGDpK7 vectors have a pK7 peptide at the C-terminus of fiber in addition to the RGD motif. Viral genomes were obtained by recombination of the corresponding pShuttle and Ad backbone plasmid in bacteria as previously described (QBiogen, Adenovator manual). All viruses were constructed and tested at the UAB Gene Therapy Center.

The lentiviral vector used in the study to obtain K562-erbB2 was constructed as described previously [32]. The plasmids for self-inactivating lentiviral vector were kindly provided by Dr. D.B. Kohn (Children's Hospital, Los Angeles). Resulting lentiviral vector contained an internal MND (Myeloproliferative sarcoma virus enhancer, Negative control region Deleted) promoter [33], human c-erbB2 cDNA and the central polypurine tract/central termination sequence. The c-erbB2 coding sequence (Gene Bank NM_004448) was amplified by PCR from the plasmid pGT36erbB2 that was kindly provided by Dr. T. Strong (UAB). The virus was generated as described by Zielske et al [32].

#### Design of transiently targeted and labeled MSC-AR

MSC-AR were obtained by transduction of MSCs with adenoviral vectors encoding the artificial receptor (AR). The artificial receptor to target MSC to ovarian carcinoma was first constructed using the pDisplay mammalian expression vector (Invitrogen, Carlsbad, CA) that allows display of proteins on the cell surface. An anti-erbB2 scFv C6.5 [34] as binding motif was fused at the N-terminus to the murine Ig κ-chain leader sequence and at the C-terminus to the platelet derived growth factor receptor (PDGFR) transmembrane domain. Recombinant AR contains the hemagglutinin A (HA) and myc epitopes for detection by Western blot. AR cDNA was then transferred to adenoviral vectors and these vectors were used to obtain MSC expressing AR on the cell membrane (MSC-AR). For all *in vitro *and *in vivo *experiments MSCs were transduced with adenoviral vectors as described previously [30] at MOI 500 vp/cell. Membrane expression of AR was confirmed by Western blot or immunohistochemistry using an anti-HA tag antibody following development with Nova-Red or DAB as HRP substrates.

MSCs expressing scFv C6.5 with anti-erbB2 specificity are labeled throughout the text as MSC-AR. MSC transduced with isogenic viral vector AdRGDpK7.GFPluc were used as an appropriate counterpart for AR-transduced cells in all experiments and labeled as MSC-GFP. Both recombinant Ad vectors (AdRGDpK7.C6.5luc and AdRGDpK7.GFPluc) for *ex vivo *MSC transduction have luciferase gene in the context of double expression cassettes: GFPluc and C6.5luc. This simultaneous loading of the transgene with a luciferase reporter allows the use of luciferase expression for quantitative comparison of MSC-AR and MSC targeting.

#### *In vitro *cell-cell interaction assays

MSC-SKOV mixed assay. Binding properties of MSC-AR were tested using SKOV3ip1 cells that abundantly express erbB2. MSCs and MSC-AR were labeled with the green fluorescent dye CFDA, whereas the SKOV3ip1 cells were labeled with the red dye SP-DiI according to the reagents' manuals. Labeled cells were lifted with Versene, washed and counted. MSCs and SKOV3ip1 were mixed in different ratios in 300 μl of PBS at 500,000 total cells/sample and incubated in solution under agitation. After washing, cell populations were separated by flow cytometry and the percentage of double-labeled cell population that corresponded to MSC-SKOV conglomerates was determined by gating on the GFP-PE population.

MSC-K562 ELISA-based assay. K562 are non-adherent cells and allow the possibility to perform ELISA-like analysis of cell-cell interaction. MSC-AR *in vitro *binding was tested using K562 cells that artificially express erbB2. K562 expressing erbB2 were obtained via lentiviral transduction. To test cell-cell interaction, the suspensions of K562 or K562-erbB2 labeled with a green fluorescent dye (CFDA) were added to MSCs or MSC-AR cultured on a plastic. After 1 hr incubation, K562 cells were washed out and all cells in the wells were trypsinized. The cell mixture was subjected to flow cytometry and the percentage of bound fluorescent cells was determined in each well. Each experimental group was assayed in triplicates.

#### *In vivo *testing of targeted MSC

Transient transgenic model. To create a state of transient expression of erbB2 in mouse lungs, we injected h-CAR transgenic mice [35] with AdCMVerbB2 i.v. Expression of the antigen in the lungs of these mice was confirmed by Western Blot of lung lysates stained with an anti-erbB2 antibody. MSC-GFP and MSC-AR labeled with firefly luciferase were injected i.v. in erbB2-preconditioned hCAR mice at 1x10^6 ^cell/mouse. Mice were followed after MSC injection by live non-invasive imaging at several time points. Upon sacrifice, luciferase expression was measured by imaging of the whole animal, imaging of the excised lungs and by luciferase expression analysis of lung lysates. To compare MSC numbers in the lungs and compensate for potential differences in luc expression in injected samples of targeted and untargeted MSC, the amount of RLU/cell was calculated for MSC-GFP and MSC-AR.

Ovarian xenograft model. Female CB17 SCID mice (Charles River, Boston, MA) 6-8 weeks of age were used to establish human ovarian xenografts. Intraperitoneal tumors were established in mice by i.p. injection of 5x10^6 ^SKOV3ip1 cells. After 14 days of tumor development, mice received intraperitoneally MSC-GFP or MSC-AR at 2x10^6 ^cells/injection. At designated time points after MSC injection, mice were subjected to non-invasive imaging for luciferase expression. The animals were subsequently sacrificed; tumor nodules, liver, spleen, kidney and part of the intestine were collected, imaged in a Petri dish and proceeded to prepare tissue lysates for conventional luciferase analysis. All visible tumor nodules in the peritoneum were collected for imaging and combined as one tumor sample for luciferase analysis.

Animal protocols were reviewed and approved by the Institutional Animal Care and Use Committee of UAB.

#### Imaging and Quantification of Bioluminescence Data

An *in vivo *optical imaging was performed with a custom-built optical imaging system with a liquid-nitrogen cooled lKB digital CCD camera (Princeton Instruments VersArray: Roper Scientific, Trenton, NJ). Mice were anesthetized with 2% isoflurane before intraperitoneal injection of d-luciferin. D-luciferin potassium salt, the substrate for firefly luciferase, was purchased from Molecular Imaging Products (Ann Arbor, Michigan). Each mouse received an injection of 2.5 mg of d-luciferin diluted in 100 μl of PBS. Mice (3 animals per group) were placed in the supine position within the imaging chamber with continuous isoflurane sedation. Whole body luminiscent images were obtained during the 5-10 min interval after injection of the substrate. Luminescence images and brightfield images were acquired with an exposure time of 60 and 0.02 sec respectively using WmView/32 software (Roper Scientific) without a filter at f/16. Index color image overlays were performed in Photoshop 7. 0 (Adobe, Seattle, W A). The range of acquisition signal was kept constant at all imaging time points. The gray scale photographic images and bioluminescence color images were superimposed using the Adobe Photoshop 7.0 software. Statistics on bioluminescent signal intensity was obtained using WinView software according to the software instruction. For comparison of tumor targeting of two cell populations, total intensity of bioluminescence signal acquired from collected tumors were normalized per tumor area. Obtained value of relative light units per area (RLU/cm2/min) is proportional to the number of cells present on tumor surfaces.

#### Luciferase expression in tissue lysates

Tumors and selected organs (liver, spleen, intestine, kidney) after imaging were used to prepare tissue lysates. Organs collected after sacrifice were homogenized using Mini Beadbeater (BioSpec Product Inc) in 500 ul of 1x tissue/cell lysis buffer (Promega). Luciferase expression in tissue lysates was determined using luciferase assay system (Promega, Madison, WI) according to the manufacturer protocol. The luciferase activities were measured in a Lumat LB 9507 luminometer (Lumat, Wallac, Inc., Gaithersburg, MD) in relative light units (RLU) and normalized by the protein concentration in cell or tissue lysates (Bio-Rad DC Protein Assay kit). To account for the potential differences in luciferase expression of the injected MSC populations (targeted and untargeted), we normalized tumor luciferase activity (RLU) by luciferase activity of the MSCs (RLU/cell) and presented data as MSC numbers per mg protein.

All *in vivo *data are presented as mean values ± standard deviation. Statistical differences among groups were analysed in a two-tailed Student's t-test using GraphPad Prizm Software (San Diego, CA).

## Results

### Design of MSC targeted to tumor markers by additional affinity

**Figure 1:**
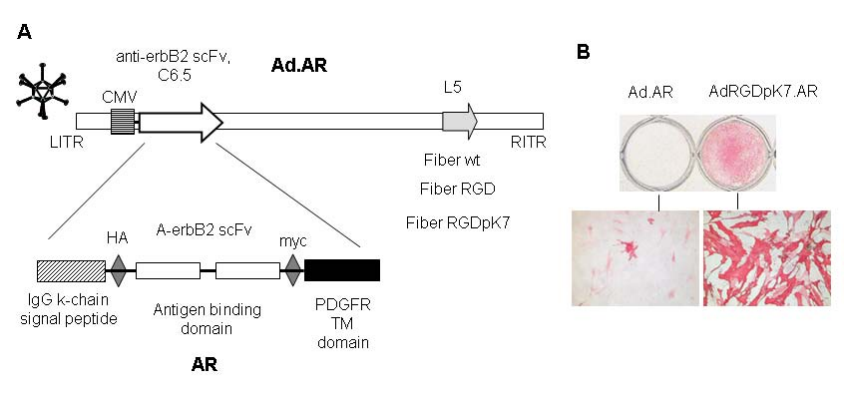
**Design of MSC-AR targeted to tumor marker**. A) Schematic presentation of Artificial Receptor C6.5 (AR) used to obtain MSC-AR and genomes of adenoviral vectors for AR expression. B) Genetically modified adenoviral vectors provided efficient expression of AR on MSC membrane. MSC were transduced with Ad.AR or AdRGDpK7.AR at MOI 500 vp/cell. AR expression on MSC membrane was confirmed by staining cells with a-HA tag antibody. MSCs expressing AR are stained red.

Although MSCs have the native ability to home to tumors, we attempted to enhance their tumor-targeting abilities by adding an additional tumor-targeting element: an artificial receptor (AR) with specificity to erbB2 (Fig. 1A). Expression of this AR on the cell membrane was obtained by transduction of cells with adenoviral expression vectors. We had previously established that adenoviruses with modified fibers have increased MSC transduction, in particular those with RGDpK7 knob modifications [12]. Thus, Ad vectors with the RGDpK7-modified fiber were constructed in this study for AR expression. The efficiency of MSC transduction by AdRGDpK7 vectors in our experimental conditions was tested by flow cytometry for GFP transgene (data not shown) and by immunostaining for AR expression (using HA expression tag). Transduction with escalating MOIs (20-500 vp/cell) resulted in progressively increasing number of infected cells. The level of 88-95% of AR-expressing cell was routinely achieved when 500 vp/cells were used (Fig. 1B), thus this MOI was then consistently used to obtain MSC-AR for all in vitro and in vivo experiments. Furthermore, for each individual experiment the levels of expression of both transgenes (AR and luc) were checked to assure that comparable cell populations were used for different experiments and to minimize the experimental variations due to variable AR expression. Although fiber-modified Ads allowed sufficient efficiency of MSC transduction, the level of AR expression on individual cell (determined as intensity of staining) was variable, which may reflect heterogeneity of MSC population as it was noted previously.

In addition, a double expression cassette incorporated into Ad vectors allowed simultaneous loading of MSCs with a targeting moiety and a reporter gene for MSC detection *in vivo*. Two Ad vectors were created for our studies: Ad.RGDpK7.**AR**.Luc and Ad.RGDpK7.**GFP**.Luc. MSC transduction with Ad.RGDpK7.**AR**.Luc enabled us to obtain erbB2-targeted luc-labeled MSCs (MSC-AR) to further test our strategy *in vitro *and *in vivo*. As an appropriate counterpart to MSC-AR, we used cells transduced with an isogenic Ad vector, in which AR gene in double cassette was substituted by GFP. These cells are labeled MSC-GFP throughout the text.

### MSC-AR bind to erbB2-expressing cells *in vitro*

**Figure 2:**
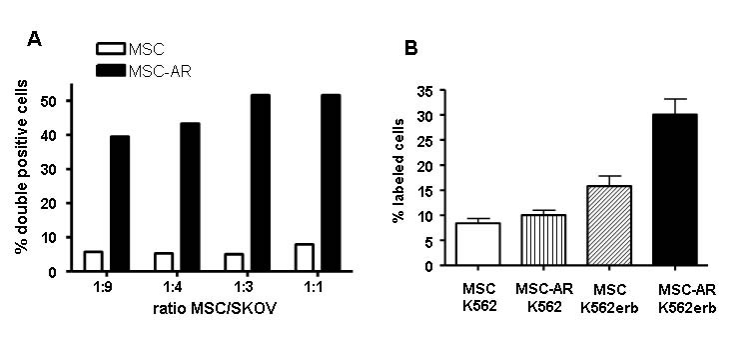
**MSC-AR bind to erbB2-expressing cells *in vitro***. A) MSC-SKOV mixed assay. MSC and MSC-AR labeled with green fluorescent dye CFDA were mixed with SKOV3ip1 cells labeled with red dye SP-DiI. After incubation in solution, cell populations were separated by FACS and percent of double-labeled population that corresponded to MSC-SKOV conglomerates was determined by gating on GFP-PE population in FACS analysis. B) MSC-K562 ELISA-based assay. K562 expressing erbB2 were obtained via lentiviral transduction. MSC or MSC-AR were cultured attached to plastic. Suspension of K562 or K562-erbB2 labeled with green fluorescent dye (CFDA) were added to cultured MSC-AR. After 1 hr incubation K562 cells were washed out and all cells in the wells were trypsinized. Cell mixture was subjected to FACS and percentage of bound fluorescent cells was determined in each well. Each group was done in triplicates.

To investigate if MSC-AR acquired new binding properties, we tested their erbB2-binding abilities *in vitro *in cell-cell binding assays. The SKOV3ip1 ovarian tumor cell line expresses high levels of erbB2, and was, therefore, used to test MSC-AR binding. MSC, MSC-AR, and SKOV3ip1 were labeled with different fluorescent dyes CFDA-CE (green) and SP-DiI (red) respectively. Both MSCs and SKOV3ip1 cells are highly adherent to plastic; therefore, binding interactions were performed in solution after cell dissociation with Versene. Cell interaction resulted in the formation of small aggregates, which were detected as a double-positive (CFDA-SP) population by flow cytometric analysis. Increasing ratios of MSC-AR: SKOV3ip1 cells consistently resulted in increased percentages of the double-positive population (39.5% at ratio 1:9, and 51% at ratio 1:3); this was in contrast to a control mixture (MSC and SKOV3ip1), where the double-positive population never exceeded 8% (Fig. 2A). Similar results were obtained in an ELISA-like assay, using nonadherent K562 cells. To render K562 cells positive for erbB2, the K562 cells were stably transduced with a lentiviral vector encoding erbB2. In this ELISA-like assay, MSCs and MSC-AR were attached to the plastic in 12-well plates to which K562 or K562-erbB2 cells labeled with a green fluorescent dye were added. After sequential washing, all cells in the wells were trypsinized and subjected to FACS analysis to determine the number of bound fluorescent cells. In accord with the previous experiment, the highest binding (30.7%) was detected in wells with MSC-AR and K562-erbB2 cells (Fig. 2B). Therefore, both *in vitro *assays confirmed that MSC-AR efficiently bind to erbB2 expressing cells.

#### MSC-AR bind to erbB2-expressing cells *in vivo*

##### Differential kinetics of MSC lung clearing in a transient transgenic model

**Figure 3:**
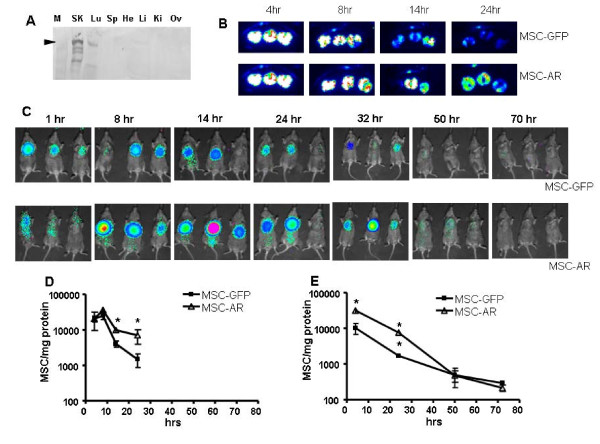
**MSC-AR bind to erbB2-expressing lung cells in vivo in transient transgenic model**. (A) Transient expression of erbB2 in the lungs of hCAR mice was induced by iv injection of AdCMVerbB2. Expression of erbB2 exclusively in the lungs of hCAR mice were confirmed by Western Blot of organ lysates. Lane M is Marker, lane SK - erbB2 positive control (SKOV3ip cell lysate), lane Lu - lung lysate, next lanes, labeled Sp, He, Li, Ki and Ov represent spleen, heart, liver, kidney, and ovary lysates correspondingly. Small triangle points to the size of erbB2-specific signal. (B-E) Kinetics of MSC-GFP and MSC-AR distribution to the erbB2-lungs of hCAR mice. MSC-GFP and MSC-AR labeled with firefly luciferase were injected iv in erbB2-preconditioned hCAR mice at 1x10^6 ^cell/mouse. In the first experiment (B, D) mice were followed at early time points after injection (4, 8, 14, 24 hrs), in the second experiment (C, E) later time points (24, 32, 50, 70 hrs) were investigated. Luciferase expression was detected by imaging of whole animal (C) imaging of excised lungs (B) and by luciferase expression analysis of lung lysates (D,E). Data are presented as number of cells per mg of protein in lung lysates. *-P = 0.05, ** - P = 0.01.

We next investigated if membrane expression of the AR could be translated to *in vivo *cell targeting advantages. We first tested our hypothesis using a transient transgenic model system, in which the erbB2 marker was artificially expressed in mouse lungs. This transient transgenic mouse model was previously used to confirm the targeting benefits of affinity-modified adenoviral vectors [36]. A transgenic mouse strain expressing the receptor for human adenovirus, hCAR [35] enables efficient infection of mouse tissues with human adenovirus. Intravenous injection of Ad vectors into the hCAR mice results in increased expression of Ad-delivered transgenes in the mouse lungs, compared to wild type C57Bl6 mice, in which 90% of the adenoviral transgene expression is detected in the liver [37,38]. This model, therefore, allows human tumor markers to be expressed in lungs, where this marker is readily accessible to systemically introduced cells. Transient expression of the erbB2 tumor marker in the lungs of hCAR transgenic mice was achieved by i.v. injection of an adenovirus encoding the erbB2 antigen (AdCMVerbB2). Expression of erbB2 exclusively in the lungs of the hCAR(+) mice compared to other organs was detected, as shown by a Western blot stained with anti-erbB2 antibodies (Fig. 3A). Injection of the same Ad vector into hCAR(-) littermates and SCID mice did not result in detectable expression of erbB2 in the lungs (data not shown). Thus, the transient transgenic model proved to be appropriate to test the effect of erbB2-lung targeting with AR-expressing MSCs.

Both MSC-GFP and MSC-AR injected i.v. first localize to the lungs due to first pass effect [39]. Thus, we did not expect to see differences at initial time points after cell injection. We wanted to evaluate differential kinetics of cells retention in the lungs as a measure of cell-cell interaction achieved by MSC-AR *in vivo *(Fig. 3B-E). Since the kinetics of this process was unknown, two experiments were carried out to investigate early (Fig. 3B, D) and late (Fig. 3C, E) time points.

The first experiment covered early time point including 4, 8, 14, 24 hrs after cell injection. Luciferase activity was measured by the intensity of the chemiluminescent signal in excised lungs and by conventional luciferase assays using whole lung lysates. To compensate for potential differences in the levels of luciferase expression in the injected samples of MSC-GFP and MSC-AR, the value of RLU/cell for each cell sample was calculated. The statistically significant differences in MSC and MSC-AR numbers were detected at 14 hrs and 24 hrs after MSC injection by both methods of luciferase detection: intensity of the lung imaging signal (Fig. 3B) and luciferase activity in lung lysates (Fig. 3D)

To trace the fate of injected cells further, we repeated the experiment including more distant time points (24, 32, 50, 70 hrs). In this experiment an increased concentration of MSC-AR in the lungs was detected starting at 8 hrs and persisted until 32 hrs after cell injection. This trend was visualized using total body images (Fig. 3C) as well as quantitatively measured by the luciferase activity in lung lysates (Fig. 3E). This effect was only transient in nature, as luciferase expression measured at the last time points (50, 72 hrs) returned to almost background levels. However, we were able to demonstrate the differences in behavior of MSC-GFP and MSC-AR injected i.v. in erbB2 expressing animals. Since both experimental groups were otherwise identical, we attribute these differences to the newly added affinity property of MSC-AR that interacted with erbB2-expressing cells.

##### Targeted MSC increase binding to erbB2-expressing ovarian tumors

**Figure 4:**
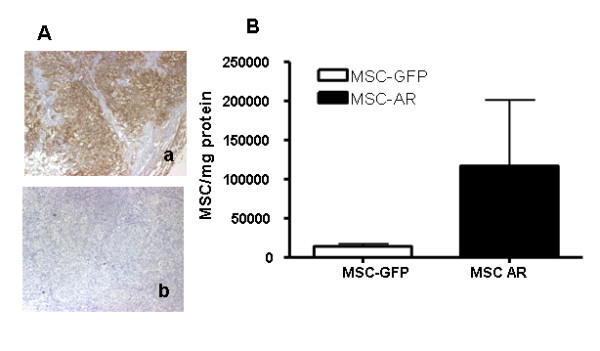
**MSC-AR increase binding to erbB2-expressing ovarian tumors**. (A) Overexpression of erbB2 in SKOV3ip ovarian tumor xenografts was confirmed by erbB2 staining (a-erbB2 staining, b-negative control). B) MSC-GFP and MSC-AR labeled with firefly luciferase were injected ip in SCID mice bearing SKOV3ip1 ovarian tumor xenografts at 2x10^6 ^cell/mouse. Tumors were collected after 24 hrs and luciferase expression was measured in luciferase analysis. Data are presented as MSC numbers per mg protein in tumor homogenates.

We have previously tested MSC homing to ovarian tumors using the SKOV3ip1 ovarian tumor xenograft model, where preferential homing of MSC to ip tumors was demonstrated, compared to other organs in the peritoneal cavity [12]. In this study we investigated if MSC concentrations in tumors are increased via expression of the tumor-specific AR on the transplanted MSCs. SKOV3ip1 ovarian tumor xenografts abundantly express erbB2, which was confirmed by erbB2 staining of tumor xenografts (Fig. 4A). Results from the previous experiment suggested that the kinetics of homing is the important parameter to investigate. Thus, we again conducted two experiments, in which the MSC numbers in tumors were assessed at different times after injection.

In a pilot experiment, tumors were collected 24 hrs after ip injection of MSC-GFP and MSC-AR and luciferase expression was measured in tumor lysates (Fig. 4B). The estimated number of MSC-AR in erbB2 expressing tumors was 117073 ± 108375 cells/mg protein, while the MSC-GFP was 14239 ± 6402 cells/mg protein. The difference the in average numbers of AR-expressing versus AR-lacking cells in tumors was substantial (8.2 folds), however, due to one tumor sample in MSC-AR group with an outstanding RLU value, it was rendered statistically insignificant by t-test analysis.

**Figure 5:**
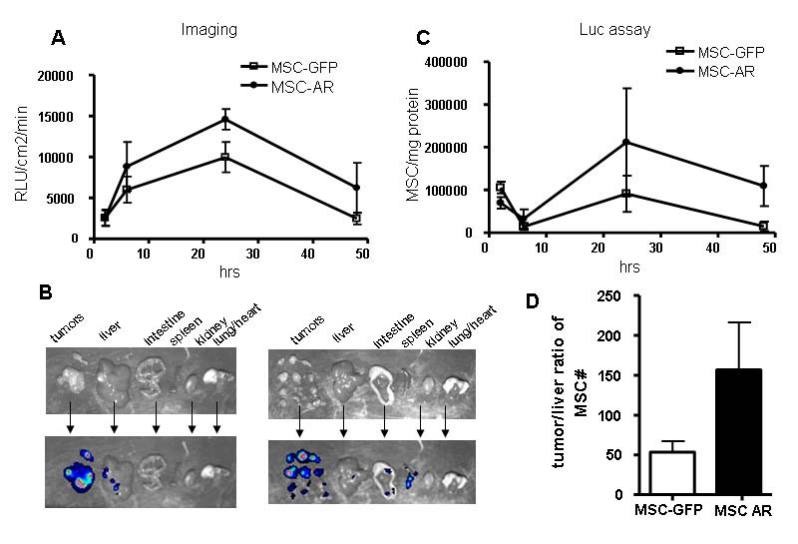
**Kinetics of MSCs homing to erbB2-expressing ovarian tumors**. MSC-GFP and MSC-AR labeled with firefly luciferase were injected ip in SCID mice bearing SKOV3ip1 ovarian tumor xenografts at 2×10^6 cell/mouse. Tumors were collected at 4, 8, 24, 48 hrs and luciferase expression was measured by imaging of excised tumors (A) and by luciferase assay of tumor lysates (C). Representative photographs of mouse organs (B, upper panel) and the same organs with overlaid bioluminescent signal (B, lower panel) are presented. Arrows connect black and white image of organ and corresponding image of the same organ with superimposed bioluminescent signal (D) Ratio of MSC numbers detected in tumor versus liver were determined for mice euthanized after 24 hrs after MSC injection. White bar is tumor/liver ratio for MSC, black bar is tumor/liver ratio of MSC-AR.

**Figure 6:**
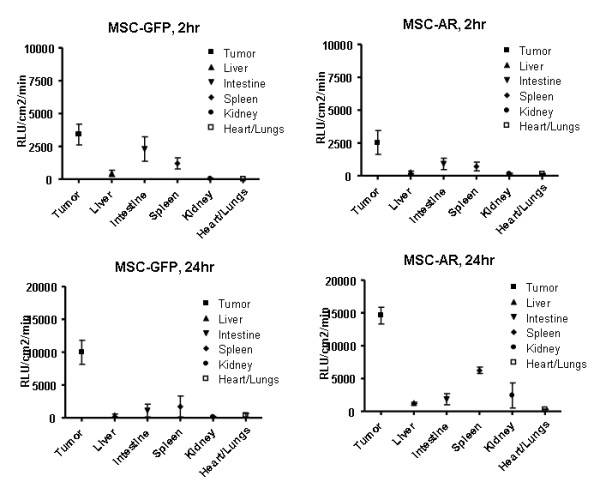
**Biodistribution of MSC-GFP and MSC-AR to the major organs in SKOV xenograft bearing mice after ip injection**. Tumors, liver, spleen, kidney, intestine, heart/lung were collected at 2 hr (upper panels) and 24 hr (lower panels) after MSC-GFP or MSC-AR injections. MSC biodistribution was detected by bioluminiscent imaging of excised organs and presented as average signal intensity per area of each organ.

To confirm this initial observation and to investigate the kinetics of cell accumulation in tumors, we conducted another experiment with a broader time line, which included evaluation of cell numbers in tumors at 2, 6, 24, and 48 hrs after MSC injection (Fig. 5). MSC-GFP and MSC-AR tumor targeting as well as biodistribution to other organs was evaluated by measuring luc expression in tumors and other major organs of the peritoneal cavity (Fig. 5 and 6). At the indicated time points we also performed whole body bioluminescent imaging, imaging of individual organs after animal sacrifice, and analysis of luciferase expression in organ lysates by conventional luc assays.

Whole body imaging typically revealed discrete zones of luciferase activity in the peritoneum, starting from the earliest time point (2 hrs) tested. The signal has a more diffuse pattern at 2 hours after cell injection, compared to the more localized pattern observed at 24 hrs in both groups (data not shown). The whole body imaging signal approximated tumor localization, however, quantitation of the signal in whole body images (thus, comparison of MSC and MSC-AR tumor homing) was not performed, as initially planned. We noted that the signal intensities in the whole body images were greatly influenced by body positioning, tumor localization in the cavity, and the extent of tumor masking by other organs.

To attribute the obtained signals to particular organs, excised tumors and organs of peritoneal cavity were imaged separately in a Petri dish. MSC biodistribution to these organs was quantitatively assessed by measuring the bioluminescent signal intensities of individual organs and luciferase activity in corresponding tissue lysates. In both groups tested, this analysis demonstrated a clear tumor preference of injected MSC (Fig. 5B). As early as 2 hrs after injection MSCs were detected in ovarian xenografts (Fig. 5A, C). Tumors were the major MSC targeting site across all time points tested in both groups, with the highest luciferase activities detected compared to the ones in other organs. This was confirmed qualitatively by detecting the luciferase signal in individual organs (Fig. 5B) and quantitatively by measuring luc activity in whole organ lysates (Fig. 6).

The intensity of the signal in tumors grew over time, reached its maximum at 24 hrs, and declined at 48 hrs (Fig. 5A, C). We did not detect differences among tumor luciferase activities in both groups at 2 and 6 hrs. However at 24 and 48 hrs, the mice that received MSC-AR demonstrated higher luciferase activity in tumors by both detection methods: tumor imaging (Fig. 5A) and luciferase activity of tumor lysates (Fig. 5C). These data are in concordance with the initial experiment, where we detected considerable differences between MSC-GFP and MSC-AR groups at 24 hrs. In the second experiment, the difference between targeted and untargeted MSC detected at this time was not as pronounced as in the pilot experiment (211744 ± 178135 and 91023 ± 84675 cells/mg protein), and corresponded only to 2.3 times difference. The means and the standard deviation range was affected by the small number of animals and individual variations between tumor samples, and rendered this difference insignificant in t-test. But, the trend of an increased number of MSC-AR in tumors was clearly detected. The increased MSC-AR numbers detected in tumors indicate increased specificity of MSC-AR tumor targeting. At early time points the MSC-GFP group showed relatively higher luciferase activities in other organs, such as spleen, liver, and intestine, compared to the organs of mice that received MSC-AR (Fig. 6). The ratio of MSC numbers in tumor versus MSC number in liver at 24 hrs was 153 for MSC-AR and 56 for MSC (Fig. 5D). Increased tumor/liver ratio indicates an increased specificity of MSC-AR tumor targeting.

## Discussion

A growing number of studies utilize engineered MSCs as a tool to track malignant tissues and deliver anticancer agents within the tumor microenvironment. MSC homing to tumors has been confirmed in a variety of experimental models, however the homing efficiency is clearly model-dependent and generally modest [3,10]. Additional cell targeting efforts may enhance the efficiency of tumor homing and consequently deliver more therapeutics. These cell targeting efforts may include physical cell routing, utilization of physiological forces for cell concentration and strategies that involve intrinsic or engineered cell homing/targeting mechanisms [40]. Targeting strategies can be used singly or in combinations to maximize cell vehicle concentration in the target site. For instance, combined native MSC tumor homing with preconditioning of the tumor site by irradiation has been shown to enhance MSC homing to irradiated tumors [41]. Native cell homing can also be combined with other types of cell targeting means [42]. The current study investigated whether native tumor targeting of MSCs can be enhanced by engineered targeting via expressing an artificial tumor-binding receptor.

Our study applied affinity-based targeting to cell vehicles that lack immune recognition. To date, only a few applications have demonstrated the feasibility of using scFvs as binding moieties in non-immune cell contexts. One example is where an artificial chimeric receptor was applied to primary human monocytes to target monocytes to CEA-expressing tumor cells [25]. Another study used gpi-anchored anti-CD20 scFv fragments exposed on red blood cells (RBC) and evaluated binding of targeted erythrocytes to CD20 positive tumors [26]. In our *in vitro *experiments, MSCs grafted with anti-erbB2 artificial receptors demonstrated increased binding to cells overexpressing erbB2 (40% in experimental group versus 8% in control). The only available study that investigated similar erbB2-based cell binding interactions [28] reported increased cell binding numbers that are in a good agreement with our results (20% in experimental group compared to 6-8% in control).

The next important question was whether the enhanced MSC-AR binding ability would translate to an *in vivo *tumor localization advantage, compared to unmodified cells. Given complexity of the processes of biodistribution and homing of injected cells, we reasoned that an effect of engineered cell targeting would be more pronounced and better detectable in model systems. For instance, an isolated heart model was used to detect the difference of MSC homing to normal versus infarcted myocardium [43]. Thus, for initial testing we choose a transient transgenic mouse model previously used to validate targeting of the affinity-modified adenoviral vectors [44]. Expression of erbB2 tumor marker in the mouse lungs ensures its easy accessibility to systemically injected cells and direct cell-marker contact. In addition, this model allows the dissection of only the affinity-related component of cell targeting, since native homing of MSC to lungs has not been reported. Of note, this model is easily manipulated whereby other markers can be tested in similar fashion.

It is not accidental that most studies detecting tumor homing of intravenously introduced MSC were performed on lung tumor models [4,10,13,45,46]. This mode of cell introduction utilizes two cell-targeting mechanisms, temporal physiological accumulation of cells in the lungs and native MSC tumor homing, whereby lung-concentrated MSCs actively migrate to local lung tumors. It was expected that accumulation of MSC in the lungs after systemic injection would be the same for modified and unmodified MSCs due to the first-pass effect [39]. However, AR-expressing cells by virtue of enhanced cell-cell interactions may show different levels of cell retention and kinetics of subsequent lung evacuation. In two subsequent experiments we have shown that MSC and MSC-AR have a different pattern of interaction with erbB2-lungs. An increased number of MSC-AR was detected in the lungs at several time points compared to MSC numbers. The time window, where the differences in experimental groups were detectable, was relatively short (14-32 hrs). At more distant time points (52, 72 hrs) MSCs were not detected in the lungs using this method. We believe that the major reason for this is MSC destruction. The hCAR transgenic mice are immunocompetent and xenogeneic (human) MSCs introduced into immunocompetent mice are likely to be killed by immune-based mechanisms over time. Despite the short window of opportunity for detecting differences, this model, nevertheless, gave us an indication that modified cells have different behavior in the model system and *in vivo *cell-cell interactions result in a detectable cell retention effect.

A more relevant and stringent model to test potential benefits of additional MSC targeting is the ovarian tumor model. We have previously demonstrated the native ability of MSC to home to SKOV3ip1 xenografts [12]. The high level of erbB2 expression makes the SKOV3ip1 model appropriate to test our double-targeting strategy, which engages both mechanisms of MSC-AR tumor targeting: native and engineered. Multiple primary and metastatic tumor nodules with generally poor developed stromal structures may again offer better accessibility of tumor markers to cell vehicles expressing AR and allows the detection of the benefit of affinity-based targeting. In the pilot experiment, a substantial increase (8-folds) in the number of tumor-associated MSC-AR versus MSC was detected. To validate this initial observation and to more accurately establish the timing of MSC homing, we investigated the kinetics of MSC tumor targeting. The speed and pattern of cell vehicles homing to target sites are important parameters to consider in designing therapeutic delivery strategies, as these values may differ considerably. For instance, homing of systemically injected CD34+ cells to bone marrow is very fast; these cells reach the bone marrow in 1 hr [47]. However, there is not much data on the efficiency and speed of MSC homing to tumors. Upon systemic injection MSC tumor homing is apparently delayed and diminished due to trapping in the lung vasculature. It is reported that upon systemic injection, MSC can stay in the lungs for two or more days [7,48], thus the intravenous route of MSC introduction is slow and inefficient. Recent quantitative studies found less than 1% of systemically injected MSC is able to reach distant sites [49,50]. In the ip tumor settings, MSCs did not have to pass the lungs, thus the anticipated time for MSC tumor homing is expected to be much shorter and efficiency better. Despite this prediction, the actual kinetics of MSC homing was unknown. In our experiment, we detected preferential homing of MSCs to tumors as early as 2 hrs, while the maximum homing of MSCs to ovarian tumors was observed at 24 hrs after cell injection. The kinetics of homing is an important parameter for our future strategy of using MSC-based vehicles to deliver oncolytic adenoviruses. Quick homing ensures that cells have enough time to reach tumors before they are killed by virus replication.

We detected preferential tumor localization of MSCs in both MSC and MSC-AR groups, which confirmed the native tumor homing abilities of these cells and assured that these functions are not perturbed by AR expression. In two separate experiments we detected an increase in the number of tumor-associated MSC-AR versus MSC-GFP starting from 24 hrs using two methods of luciferase quantitation.

The analysis of MSC distribution in the peritoneum demonstrated that intraperitoneally developed tumors are the major cell homing sites in both groups and across all time points tested. The ability to localize to even minor tumor metastasis at the surface of organs is a remarkable property of MSCs that can be exploited for diagnostic or treatment endpoints. Although addition of the AR might not influence the actual process of MSC moving towards a chemotactic homing gradient, it was able to "strengthen" binding to tumors and resulted in increased tumor-associated cell numbers and the tumor/liver ratio. The mechanism of these effects is potentially mediated by increased adherence to tumor cells, which affected both the efficacy (number of cells attached to tumors) as well as the specificity (ratio of tumor-bound versus other organ bound cells).

In both models we capitalized on the accessibility of targeting tumor markers to cells expressing AR. The necessity of the accessibility may dictate a careful selection of the marker and corresponding targeting moiety. For instance, under systemic injection, circulated cells are most likely to have physical contact with endothelial cells, supporting the targeting of specific endothelium markers. Such selective binding of circulating cells to key neovasculature markers has been described [51,52]. An avenue for utilizing tumor markers may be facilitated by irregular and atypical tumor vasculature allowing direct contact of circulating cell vehicles with tumor cells. Further studies to identify such markers and to test if cell targeting to these markers increases their retention in tumors are needed.

Another important issue concerning the therapeutic use of cells as vehicles is the quantity of cells reaching the desired destination, as well as an understanding of how these numbers translate to therapeutic benefits. Some applications might need a maximum possible cell number to achieve a therapeutic benefit [6,7,53], while others may benefit from delivery just a few cells to trigger the desired effect [45]. Thus, knowledge of the quantitative characteristics of cell homing in different models is useful and needed for further translation of these strategies to the clinic.

The majority of studies exploiting MSC tumor homing have only demonstrated the presence of labeled MSCs in the tumor parenchyma [8,11,13,29]. The quantitative aspect of cell homing or targeting to some extent is present in the available literature, however, it is not usually the major subject of these studies and, therefore, is not systematically approached. Meaningful information on homing efficiency can only be extracted when comparisons are performed within the same study. For instance, such comparisons are reported on different routes of MSC injection [54] or homing of MSC to non-irradiated versus irradiated tumors in single animal [41,55] or comparing MSC and 3T3 tumor homing [9].

Among the studies that attempted quantitation of MSC numbers in particular sites, only moderate cell numbers in the targeted tissues were reported [3,10]. MSC numbers in these studies are mostly expressed as relative units, thus, preventing the calculation of the actual homing efficiencies as a proportion of the injected cell dose. To date, most studies attempting MSC quantitaion were performed on lung tumors or lung metastases. Despite moderate cell numbers in lung tumors reported, these studies demonstrated the therapeutic effect of local cell-based transgene delivery. This is an important observation, as it demonstrates that even moderate cell numbers in lung tumors are sufficient to show a therapeutic benefit to this approach. Studies investigating MSC homing to distant (subcutaneous) tumors after systemic injection reported more controversial numbers [5]. While the presence of MSCs in subcutaneous tumors after iv introduction in general was demonstrated, only one study has reported on the therapeutic efficacy of MSC-based delivery to sc tumors [8]. The benefits of therapeutic treatment in such settings remain to be proved. Thus, despite the fact of the recruitment of MSC to tumors has been established in a variety of experimental models, the efficacy of this process in each case varies and is still presents a subject for investigation.

The major purpose of our study was to investigate the differences that AR-modified cells achieve in tumor targeting. Therefore, thorough quantitative evaluation of absolute cell numbers per tumor or other organs was not performed. Of note, some features of the ovarian tumor model influence accurate quantitative estimation of tumor cell homing and have to be accounted for. Multiple tumor nodules and metastasis hamper accurate collection of the entire tumor sample, which results in underestimation of total MSC tumor homing levels. On the contrary, metastases to organs (spleen, liver, intestine surfaces), if not identified and dissected out, may incorrectly attribute MSC homing to these organs, especially using bulk assays such as luciferase activity of organ lysate. This leads to an overestimation of MSC distribution to off-tumor sites, while, in fact, this is also tumor-related homing. Therefore, accurate quantitative analysis would require more attention to both, the procedure of organ collection for analysis, and the ability to better visualize tumor nodules.

Nevertheless, the importance of quantitative assessment, as well as developing an accurate methodology for determination of absolute cells numbers in organs has been recognized. Thus, tumor homing data were presented as cells per mg of protein, which gives a ballpark estimation of cell numbers present in tumors at these conditions. The level of native MSC tumor targeting was roughly estimated as 10% of the injected cell dose, while addition of the artificial receptor increased this efficiency 1.5-2 times. Based on this estimation, we believe that MSC-based therapeutic delivery has more practical utility after tumor debulking. In the residual disease it may provide much higher vehicle/tumor cell ratio than cases in which large primary tumors exist. Also, a marker targeted by MSC-AR will be better exposed on small tumor nodules without prominent stromal component or on patches of disseminated tumor cells.

## Conclusions

Our study confirmed that modification of cell carriers via expressing artificial receptor mediates the numbers of injected cell carriers in the organ or site of interest. We have demonstrated the practical relevance of our strategy in an ovarian tumor model, and showed that the number of modified MSC carriers increased in intraperitoneal tumors. Artificial receptor strategy can be applicable to other cell types, especially to circulating cells lacking native homing abilities.

## Competing interests

The authors declare that they have no competing interests.

## Authors' contributions

SK, LP carried out all study including in vitro and in vivo experiments needed to test porposed strategy. JR helped with in vitro studies, drafting and editing the manuscript. RA carried out general supervision regarding ovarian model used, involved in drafting the manuscript. DTC oversaw the project, have made contribution to study design and discussion of ideas and results. LP have made the major contribution to developing the concept of cell targeting, carried out all study design, acquisition and interpretation of data, wrote and edited the manuscript. All authors read and approved the final version of the manuscript.
